# Female Representation in Academic Medicine in Pakistan: A 15-Year Overview

**DOI:** 10.7759/cureus.26210

**Published:** 2022-06-22

**Authors:** Samina Ismail, Fiza Khan, Malika Hameed

**Affiliations:** 1 Anaesthesiology, Aga Khan University, Karachi, PAK; 2 Anaesthesiology, Aga Khan University Hospital, Karachi, PAK

**Keywords:** scientific publications, pakistani authors, anaesthesia, developing countries, academic contribution, academic medicine, female researchers, female authors

## Abstract

Objective: To determine the proportion of female authors publishing in Pakistan and their representation in academic anesthesiology*. *

Design, place, and duration of study: This study was a cross-sectional retrospective analysis. We reviewed all volumes and issues of the Journal of the College of Physicians and Surgeons Pakistan (JCPSP) published from 2007 to 2021. All original articles, clinical practice articles (CPAs), reviews, and editorials were included. The first and last authors publishing in JCPSP were the study subjects.

Main outcome measures: Gender of the first and last authors was determined by (a) a general review of the author’s first, middle, and last names, (b) an internet search of the author’s name, and a review of photographs on their social media, or (c) an online search of the author’s first name for typical gender assignment. The research field of the first author was noted to determine the contribution of different medical specialties. Article type and the number of citations were noted to determine the relationship with the gender of the author.

Results: Around 1549 papers were published by Pakistani authors, of which, 82.6% were original articles, 9.8% were editorials, 5.5% were CPAs, and 2.1% were reviews. Around 56.2% of the first authors and 70.9% of the last authors were males. Most article types had a majority of male first and last authors (<0.001). The median (interquartile range) citation rate was two (0-19), with no difference in citations between gender. Male-male author pairing remained the most common (45.6%). The majority of the papers published belonged to the field of medicine (27.2%) and surgery (21%), with only 3.1% contributed by anesthesiology (females: 41.3%; males: 58.6%).

Conclusion: Female representation in academia in Pakistan is at par with developed countries. The academic contribution from anesthesiology remains low, which corresponds to a lower percentage of the anesthesia workforce in the country. There is a need for a national indexed journal of anesthesia to evaluate the true representation of female authors in the country.

## Introduction

For decades, the field of medicine has been biased by male representation. Although female representation in clinical capacity is increasing [1], they remain underrepresented in leading academic positions [2]. Multiple sociological and structural barriers have been reported worldwide that hinder female advancement academically in the field of medicine [3-5]. These reasons are expected to be more in a country like Pakistan with cultural constraints and Islamic influence.

As scientific publications serve as an essential metric for academic advancement, developed countries are proactive to publish gendered publishing trends in anesthesia and other medical specialties [6-10]. However, no such data are available from developing countries, including Pakistan, where the representation of female authors in medicine remains unknown.

The Journal of the College of Physicians and Surgeons Pakistan (JCPSP) is the official journal of the College of Physicians and Surgeons Pakistan (CPSP), an institution responsible for certifying clinicians in the country since 1962. It is a PubMed-indexed, multidisciplinary journal receiving articles from all medical specialties including anesthesia. As academic productivity mainly depends on indexed publications, it is imperative to assess the current role of female authors from an indexed journal in Pakistan. With JCPSP being the official journal of CPSP and a popular option for publishing among clinicians working in Pakistan, we believe it to be an accurate source to explore the role of female Pakistani researchers, clinicians, and anesthesiologists.

The primary objective of this retrospective analysis is to determine the proportion of Pakistani female first and last authors in original articles, clinical practice articles (CPAs), reviews, and editorials published in the last 15 years in JCPSP. The secondary objective is to assess female representation in JCPSP in the field of anesthesiology from Pakistan.

## Materials and methods

In this cross-sectional retrospective analysis of the first and last authors’ gender from articles published in JCPSP, we reviewed all volumes and issues of the journal published from 2007 to 2021 (15 years). All original articles, CPAs, reviews (narrative, systematic, and meta-analysis), and editorials published by Pakistani authors, determined from affiliations, were included. Other article types (e.g. letters to the editor, clinical case reports, audits, and evidence-based reports) and papers published by international authors were excluded. Ethical Research Committee’s (ERC) approval was not required for this study as only publicly available data were extracted and included from the journal’s website (https://jcpsp.pk/archive.php) and the internet. However, permission was sought via email from the editorial board of JCPSP before the commencement of the data extraction. Our focus remained on the first and last authorships as these hold the most weightage in academic promotion, with the first authors playing the role of principal investigators while the last one publishing in a supervisory role [11].

To facilitate the data extraction, a data extraction form was designed using Research Electronic Data Capture (REDCap) electronic data capture tool hosted at the Aga Khan University [12,13]. The data extraction form included the year of publication, gender of the first author, gender of the last author, country of origin, the research field of the first author, article type (original, CPA, review, and editorial), and the number of citations. Sex is defined by biological attributes, mostly assigned at birth, based on genetic composition and genitalia, that separate males from females while gender refers to socially constructed identities and roles that may be classified as feminine and masculine [14]. Gender was assigned to the authors using one of the following approaches: (a) general review of the author’s first, middle, and last names (most used approach); (b) internet search of the author’s name and review of photographs on their social media (ResearchGate, LinkedIn, or Twitter); (c) lastly, online search of the author’s first name for typical gender assignment (https://www.genderchecker.com/). If gender was not established using one of these approaches, they were coded as "cannot be identified". When an article was authored by a single author, the author’s gender was included as the first author’s gender. Articles where the gender of the first author could not be identified were excluded from the analysis.

The country of origin and medical specialty of the first author were identified by reviewing the author affiliation mentioned in the article. To feasibly report the magnitude of contribution made by authors from different medical specialties in JCPSP, we created the following medical specialty categories: medicine (all medical subspecialties, including critical care by the medicine department), surgery (all subspecialties), anesthesiology (including intensive and critical care), radiology, pathology (histology/microbiology), emergency medicine, dentistry, pediatrics, obstetrics and gynecology, and the miscellaneous group. All articles published by authors affiliated with basic sciences (pharmacology, biochemistry, anatomy, and physiology), epidemiology and public health, biotechnology and animal research, psychology, lab-based research, and medical education were classified into a miscellaneous group. As the JCPSP did not report the number of citations on the journal website, these were obtained from PubMed (https://pubmed.ncbi.nlm.nih.gov/).

The proportion and gender distribution of doctors and dentists currently registered in Pakistan were ascertained to draw comparisons with the proportion of publishing authors in the country. These data were obtained by approaching the Pakistan Medical Commission (PMC) office, with a special request to the PMC Chair, by the primary investigator via email and phone, as data from 2021 were not yet updated on the website (https://www.pmc.gov.pk/).

Data were analyzed using RStudio 4.1.3 (R Foundation for Statistical Computing, Vienna, Austria). Descriptive statistics were computed to describe data, with qualitative variables (country, article type, gender, and medical specialty) presented as frequencies or percentages (%) and citations (quantitative variable) presented as median with interquartile range (IQR). To compare the proportion of male and female authors for each article type, the Z-score test was used. Wilcoxon rank-sum test was used to assess the difference between median citations for the gender of first and last authors. A p-value < 0.05 was considered significant.

## Results

On screening the JCPSP archives from 2007 to 2021, we identified a total of 1547 articles that met our inclusion criteria. Of these, the majority were original articles (82.6%), followed by editorials (9.8%), CPAs (5.5%), and reviews (2.1%). Authors belonged to various medical specialties with a low representation from the field of anesthesia (1.87%, 29/1547). The majority of the authors were affiliated with the fields of medicine and medical intensive care (N = 421, 27.2%) and surgery (N = 326, 21%), followed by other specialties including pathology (N = 186, 8.4%) and pediatrics (N = 109, 7%), radiology (N = 83, 5.3%), obstetrics and gynecology (N = 81, 5.2%), dentistry (N = 62, 4%), and emergency medicine (N = 4, 0.25%). In addition, the miscellaneous group contributed 16% of the articles (N = 246).

Gender distribution of Pakistani authors

The proportion of male first and last authors was significantly higher when compared to female first (43.7% vs. 56.2%, p < 0.001) and last authors (29.1% vs. 70.9%, p < 0.001) (Table 1). Similarly, most of the original articles, editorials, and CPAs were authored by male first authors when compared with females with a significant difference seen in original articles (<0.001) and editorials (<0.001). Although not significant, women authored the majority (51.5%, 17/33) of the review articles as the first authors. The proportion of male authors was significantly higher for all article types for the last authorship positions (Table 1). The proportion of first and last male authors remained consistently high for all article subtypes over time (Supplements A and B).

**Table 1:** Relationship between author gender with the type of article and citation rate

	First author			Last author		
	Female	Male	P-value	Female	Male	P-value
All articles, n (%)	677/1547 (43.7)	870/1547 (56.2)	<0.001	411/1412 (29.1)	1001/1412 (70.9)	<0.001
Original articles, n (%)	571/1277 (44.7)	706/1277 (55.3)	<0.001	353/1229 (28.7)	876/1229 (71.3)	<0.001
Editorials, n (%)	47/152 (30.9)	105/152 (69.1)	<0.001	26/67 (38.8)	41/67 (61.2)	0.009
Clinical practice articles, n (%)	42/85 (49.4)	43/85 (50.6)	0.857	21/85 (24.7)	64/85 (75.3)	<0.001
Reviews, n (%)	17/33 (51.5)	16/33 (48.5)	0.8103	11/31 (35.5)	20/31(64.5)	0.023
Citation rate, median (interquartile range)	2 (0, 6)	2 (0, 6)	0.247	2 (0, 6)	2 (0, 6)	0.360

**Figure 1:**
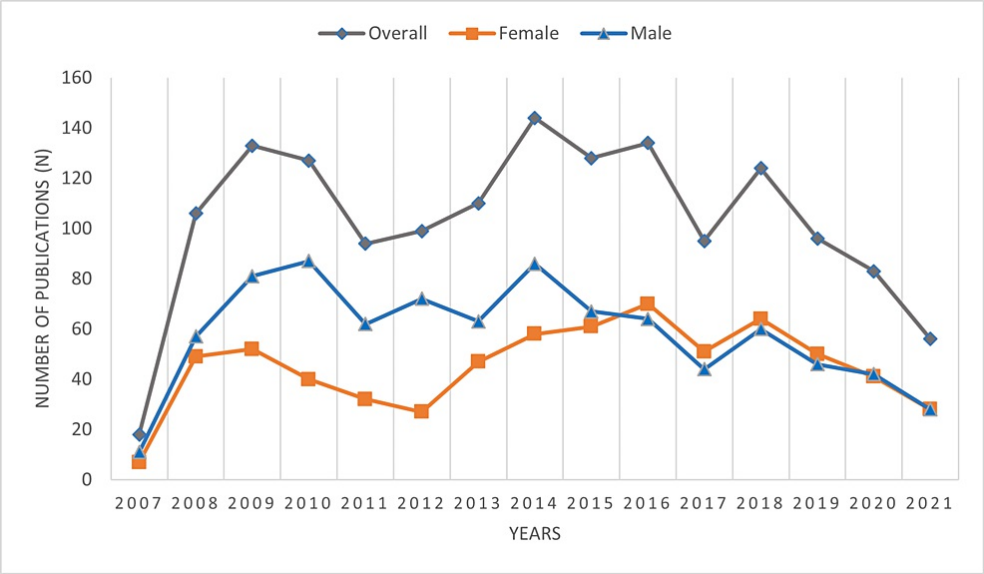
Authorship trends with respect to gender over time

The trend of male and female first authorships remained fluctuant across the past 15 years. From 2009 to 2015, there was a wide difference in the proportion of male and female first authors with the male contribution being predominant. However, this trend changed in the last six years (2015-2021), where more females published as first authors when compared to their male colleagues with female first authors superseding male first authors in 2016 (52.2% vs. 47.7%). The contribution of females’ first authorships equaled that of males in 2021 (N = 28 vs. N = 28, respectively) (Figure 1).

Relationship between Pakistani authors’ gender and citation rates

On average, articles have been cited a median (IQR) of two (0-6) times with no difference in citation rate noted between all article types (Supplement C). Similarly, no difference in the number of citations was observed for male and female first and last authors (Table 1).

Pairing of authors from Pakistan over time

**Figure 2:**
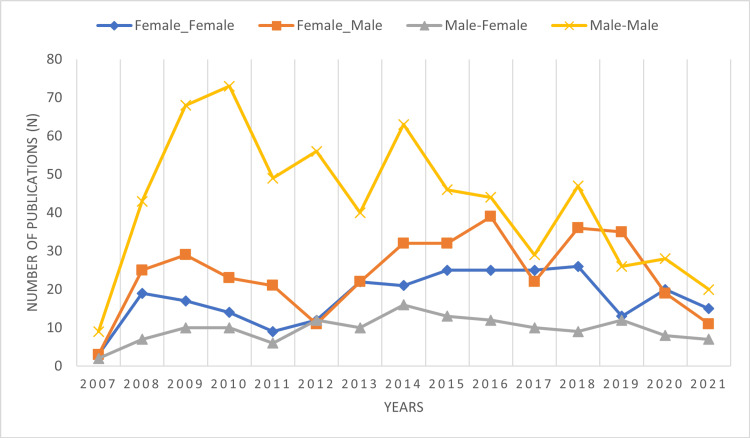
Male and female authorship pairing overtime

Of the total 1411 (91.2%) papers having more than one author, the majority (N = 641, 45.6%) of articles had both male first and last authors, 35.7% (N = 504) had mixed pairing, and less than one-fifth (N = 266, 18.8%) had a female-only pairing. For mixed pairing, the majority of female first authors paired with male last authors (71.4%, 360/504) while only 28.6% (144/504) male first authors paired with female last authors (Supplement D). These pairings remained fluctuant over time; the latest data from 2021 show a decreasing trend of female-male (N = 11) author pairing when compared to the female-female (N = 15) pairing (Figure 2, Supplement E).

Representation of Pakistani female authors in different medical specialties

**Figure 3:**
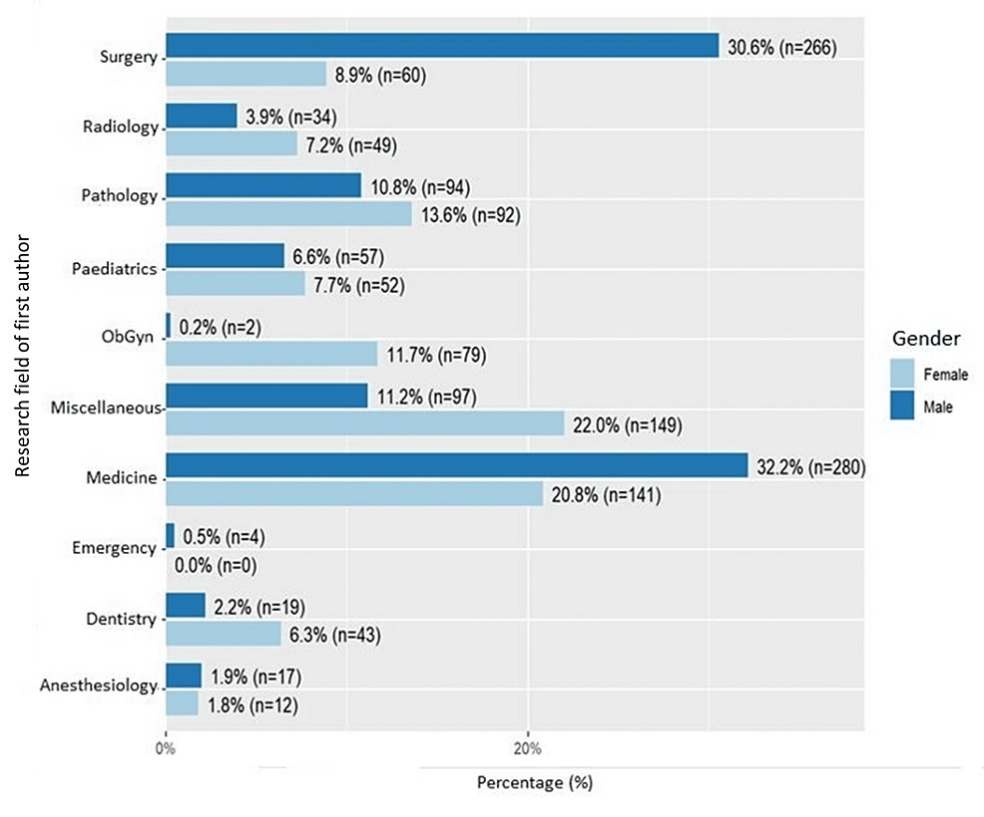
Proportion of male and female first authors in different medical research fields

Pakistani authors mostly belonged to the field of medicine (first author: n = 421, 27.2%; last author: n = 392, 27.7%) and surgery (first author: n = 326, 21%; last author: n = 313, 22.16%). The gender distribution among different research fields is highlighted in Figure 3. Although male first authors contributed to most articles published in medicine (n = 280 vs. n = 141 females) and surgery (n = 266 vs. n = 60), female first authors dominated in all other specialties. The highest female representation was noted in the miscellaneous category (n = 148 vs. n = 97 males), followed by pathology, obstetrics/gynecology, pediatrics, and dentistry. Of the first authors publishing in anesthesiology, 41.3% were females while 58.6% were males.

**Figure 4:**
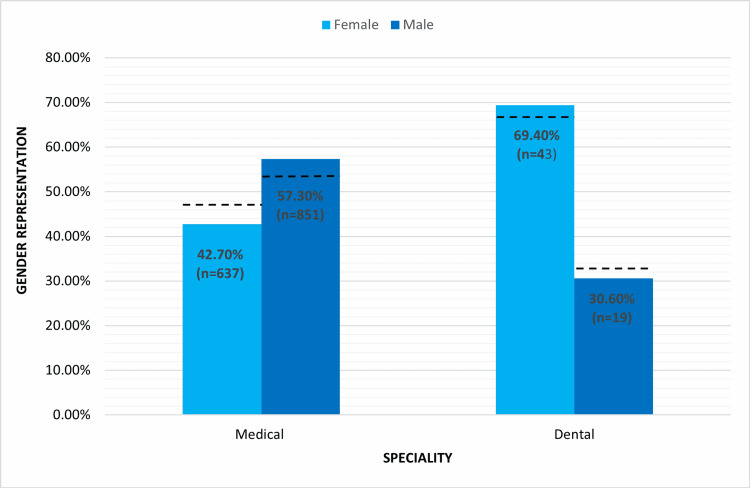
Proportion of female and male first authors in medicine and dentistry The dotted line represents the proportion of female and male doctors and dentists in Pakistan.

When comparing research published by first authors from medical and dentistry backgrounds, it was found that almost 70% of the first authors in dentistry were females while 42.7% of first authors in medical specialties were females. As per the PMC data, a total of 271,560 doctors (females = 46.9% and males = 53.1%) and 31,867 (females = 66.5% and males = 33.5%) dentists are currently registered with the PMC (Supplement F). The proportion of male to female doctors and dentists practicing in Pakistan corresponded to the proportion of male and female authors in their respective fields in the country (dotted line, Figure 4).

According to the recent data obtained from the PMC office, the total number of registered anesthesiologists in Pakistan is 3488 and those registered with a specialist qualification (Fellow of College of Physicians and Surgeons (FCPS) Anesthesiology, Fellowship of the Royal College of Anaesthetists (FRCA), or Diplomate American Board of Anesthesiology) in the field of anesthesia are 1186 (active license = 1142 and inactive license = 44). When compared to the total number of doctors registered with PMC, the percentage of the registered anesthesiologist is 12.6% (3488/271,560) and the percentage of specialist anesthesiologists with active licenses is 0.42% (1142/27,150). Of the anesthesiologists with a specialist qualification and an active license, 73.2% (n = 836/1142) are males while 26.7% (n = 306/1142) are females. For the first authors publishing in anesthesiology, we found that 41.3% were females while 58.6% were males. When comparing the proportion of female anesthesiologists to the female first authors in anesthesiology, we found that the proportion of female authors in anesthesiology appears to outpace the proportion of female anesthesiologists (41.3% vs. 26.7%) in Pakistan.

## Discussion

The findings from our review suggest that the majority of articles (73%) published in JCPSP are from Pakistani authors and have a high male representation (56.2%). However, a changing trend is observed as female authors have either equaled or superseded their position as first authors in publications in the last six years (2015-2021). Female representation as authors was higher in all medical specialties other than medicine, surgery, and anesthesia. As major contributions of research papers were from medicine and surgery, an overall high male author representation was observed in our study.

The underrepresentation of females in academic medicine is not a problem unique to a developing country like Pakistan. Our finding of low female representation as authors is in concordance with the data from developed countries [6,9,10,15]. As Pakistan lacks an indexed journal specific to anesthesia and JCPSP is a multidisciplinary journal, we found low research contribution from the field of anesthesiology (3.1%). This low representation corresponds to an overall low percentage of anesthesiologists (12.6%) compared to the total number of doctors registered with PMC. Out of specialist anesthesiologists, licensed in Pakistan, 3.8% are females. Our data show that 41.3% of the first authors publishing in the field of anesthesiology in JCPSP were females. Therefore, when comparing the proportion of female anesthesiologists in Pakistan to female first authors publishing in JCPSP, we found that the proportion of female authors appears to have outpaced the proportion of female anesthesiologists (Supplement B). Data from journals that are specific to anesthesia, such as the Canadian Journal of Anesthesia [6,8], Anesthesiology, and Anesthesia & Analgesia [6], quote female representation as 19%, 32%, and 30%, respectively.

Trends of lower female contribution in research are not only specific to anesthesiology [6] but also in other medical subspecialties as well, such as pediatrics [11] and gastroenterology [16]. Our analysis of the proportion of male to female first authors ratio in other medical specialties revealed a major difference in the specialty of surgery (81.5% males vs. 18.4% females) followed by medicine (66.5% males vs. 33.4% females). In all the remaining specialties like obstetrics and gynecology, pediatrics, pathology, radiology, dentistry, and miscellaneous group, female representation as first authors was found to be higher than their male counterparts.

The encouraging finding in our study was to see an increase in the number of females as first authors in the last six years. This finding is in concordance with the worldwide increase in the proportion of women in medical science [17-21], which in turn is associated with a higher representation of women authoring scientific papers [7,10,22]. The past several decades of persistent underrepresentation of women in science and medical fields [23] has prompted the launch of programs to improve the participation and advancement of women in academic careers [24]. The recent increase in female authorship in scientific publications may be attributed to these initiatives.

The author’s position on the article by-line depends on the contribution to research projects as well as seniority and overall responsibility in the overall work [25]. In our study, we observed more males (64.2%) compared to females as the last authors. However, Jagsi et al. [7] reported an increase in the proportion of women at the last position in scientific papers for over 30 years period.

Our analysis showed that male senior authors are more likely to collaborate with male first authors than female first authors (45.42% vs. 25.51%). As far as senior female authors are concerned, they mentor and collaborate with both male and female first authors (10.20% vs. 18.8%). Mentorship is reported to have a positive influence on personal development, career guidance, research productivity, and success. Mentors help in the development of self-confidence and provide support and resources for research activities [26]. Women in medicine perceive that they have more difficulty in finding mentors than their male colleagues [26]; however, we have noticed in our study a rising trend of females mentoring females.

The major strength of our study is that it has given an overview of female representation as authors from a developing country. The available literature is mostly from the developed world, which is not a true representation of females as researchers and authors from developing nations like Pakistan. Several recent articles have attempted to study the socio-cultural norms around gender and work in Pakistan [27,28], including the issues of “bride doctors” where females from Pakistan quit practice after getting married [29,30]. Therefore, the issue concerning low female representation in academia is much more serious in Pakistan than in developed countries.

Our study also has some limitations. Firstly, we selected and assessed female representation from a single medical journal, JCPSP, published in Pakistan. The main reason for selecting JCPSP is because it is the official journal of CPSP and due to this journal’s reputation and popularity in the country, the medical professionals were more biased toward the publication of their research in the journal associated with CPSP. The second limitation is that due to the multidisciplinary nature of JCPSP, the true representation of female contribution to anesthesia cannot be ascertained. As Pakistan does not have an indexed journal for anesthesiology, most researchers submit their papers to indexed multidisciplinary journals of the country or to international journals.

## Conclusions

Overall, our study demonstrates that female representation in a medical journal from Pakistan is at par with developed countries. Despite higher male representation as authors in JCPSP, there is an overall increasing trend of female representation in recent years. Overall, there is a small contribution of articles from the field of anesthesiology, which is probably due to the lower percentage of the anesthesia workforce in the country compared to other specialties. There is a need for a separate PubMed-indexed journal for the specialty of anesthesia to evaluate the true representation of female anesthesiologists in academia.

## Appendices

Supplement A

**Table 2:** Types of articles and gender of the first author with the year of publication

	Clinical practice articles		Editorials		Original article		Reviews		Overall	
Publication year	Female (N = 42) (%)	Male (N = 43) (%)	Female (N = 47) (%)	Male (N = 105) (%)	Female (N = 571) (%)	Male (N = 706) (%)	Female (N = 17) (%)	Male (N = 16) (%)	Female (N = 677) (%)	Male (N = 870) (%)
2007	0 (0)	0 (0)	2 (4.26)	0 (0)	5 (0.876)	11 (1.56)	0 (0)	0 (0)	7 (1.03)	11 (1.26)
2008	0 (0)	0 (0)	2 (4.26)	6 (5.71)	47 (8.23)	51 (7.22)	0 (0)	0 (0)	49 (7.24)	57 (6.55)
2009	0 (0)	1 (2.33)	8 (17.0)	5 (4.76)	43 (7.53)	75 (10.6)	1 (5.88)	0 (0)	52 (7.68)	81 (9.31)
2010	1 (2.38)	1 (2.33)	4 (8.51)	5 (4.76)	35 (6.13)	81 (11.5)	0 (0)	0 (0)	40 (5.91)	87 (10.0)
2011	2 (4.76)	2 (4.65)	1 (2.13)	8 (7.62)	29 (5.08)	52 (7.37)	0 (0)	0 (0)	32 (4.73)	62 (7.13)
2012	1 (2.38)	1 (2.33)	5 (10.6)	7 (6.67)	21 (3.68)	62 (8.78)	0 (0)	2 (12.5)	27 (3.99)	72 (8.28)
2013	0 (0)	1 (2.33)	2 (4.26)	9 (8.57)	44 (7.71)	53 (7.51)	1 (5.88)	0 (0)	47 (6.94)	63 (7.24)
2014	2 (4.76)	2 (4.65)	1 (2.13)	10 (9.52)	54 (9.46)	73 (10.3)	1 (5.88)	1 (6.25)	58 (8.57)	86 (9.89)
2015	2 (4.76)	2 (4.65)	2 (4.26)	10 (9.52)	56 (9.81)	52 (7.37)	1 (5.88)	3 (18.8)	61 (9.01)	67 (7.70)
2016	0 (0)	2 (4.65)	4 (8.51)	8 (7.62)	64 (11.2)	52 (7.37)	2 (11.8)	2 (12.5)	70 (10.3)	64 (7.36)
2017	1 (2.38)	3 (6.98)	4 (8.51)	8 (7.62)	43 (7.53)	31 (4.39)	3 (17.6)	2 (12.5)	51 (7.53)	44 (5.06)
2018	7 (16.7)	5 (11.6)	5 (10.6)	7 (6.67)	50 (8.76)	44 (6.23)	2 (11.8)	4 (25.0)	64 (9.45)	60 (6.90)
2019	9 (21.4)	4 (9.30)	1 (2.13)	11 (10.5)	38 (6.65)	29 (4.11)	2 (11.8)	2 (12.5)	50 (7.39)	46 (5.29)
2020	12 (28.6)	12 (27.9)	4 (8.51)	7 (6.67)	22 (3.85)	23 (3.26)	3 (17.6)	0 (0)	41 (6.06)	42 (4.83)
2021	5 (11.9)	7 (16.3)	2 (4.26)	4 (3.81)	20 (3.50)	17 (2.41)	1 (5.88)	0 (0)	28 (4.14)	28 (3.22)

Supplement B

**Table 3:** Types of articles and gender of the last author with the year of publication

	Clinical practice articles		Editorials		Original article		Reviews		Overall	
Publication year	Female (N = 21) (%)	Male (N = 64) (%)	Female (N = 26) (%)	Male (N = 41) (%)	Female (N = 353) (%)	Male (N = 876) (%)	Female (N = 11) (%)	Male (N = 20) (%)	Female (N = 411) (%)	Male (N = 1001) (%)
2007	0 (0)	0 (0)	1 (3.85)	0 (0)	4 (1.13)	12 (1.37)	0 (0)	0 (0)	5 (1.22)	12 (1.20)
2008	0 (0)	0 (0)	0 (0)	1 (2.44)	26 (7.37)	67 (7.65)	0 (0)	0 (0)	26 (6.33)	68 (6.79)
2009	0 (0)	1 (1.56)	3 (11.5)	3 (7.32)	23 (6.52)	93 (10.6)	1 (9.09)	0 (0)	27 (6.57)	97 (9.69)
2010	2 (9.52)	0 (0)	1 (3.85)	4 (9.76)	21 (5.95)	92 (10.5)	0 (0)	0 (0)	24 (5.84)	96 (9.59)
2011	0 (0)	4 (6.25)	1 (3.85)	3 (7.32)	15 (4.25)	63 (7.19)	0 (0)	0 (0)	16 (3.89)	70 (6.99)
2012	0 (0)	2 (3.13)	1 (3.85)	3 (7.32)	22 (6.23)	61 (6.96)	1 (9.09)	1 (5.00)	24 (5.84)	67 (6.69)
2013	1 (4.76)	0 (0)	1 (3.85)	4 (9.76)	30 (8.50)	57 (6.51)	0 (0)	1 (5.00)	32 (7.79)	62 (6.19)
2014	2 (9.52)	2 (3.13)	1 (3.85)	3 (7.32)	34 (9.63)	88 (10.0)	0 (0)	2 (10.0)	37 (9.00)	95 (9.49)
2015	0 (0)	4 (6.25)	2 (7.69)	4 (9.76)	35 (9.92)	68 (7.76)	1 (9.09)	2 (10.0)	38 (9.25)	78 (7.79)
2016	0 (0)	2 (3.13)	1 (3.85)	3 (7.32)	35 (9.92)	75 (8.56)	1 (9.09)	3 (15.0)	37 (9.00)	83 (8.29)
2017	2 (9.52)	2 (3.13)	5 (19.2)	2 (4.88)	26 (7.37)	44 (5.02)	2 (18.2)	3 (15.0)	35 (8.52)	51 (5.09)
2018	4 (19.0)	8 (12.5)	1 (3.85)	6 (14.6)	28 (7.93)	66 (7.53)	2 (18.2)	3 (15.0)	35 (8.52)	83 (8.29)
2019	2 (9.52)	11 (17.2)	4 (15.4)	1 (2.44)	19 (5.38)	45 (5.14)	0 (0)	4 (20.0)	25 (6.08)	61 (6.09)
2020	6 (28.6)	18 (28.1)	2 (7.69)	2 (4.88)	17 (4.82)	27 (3.08)	3 (27.3)	0 (0)	28 (6.81)	47 (4.70)
2021	2 (9.52)	10 (15.6)	2 (7.69)	2 (4.88)	18 (5.10)	18 (2.05)	0 (0)	1 (5.00)	22 (5.35)	31 (3.10)

Supplement C

**Table 4:** Median citation for the type of articles and gender IQR, interquartile range.

Article type		First author		Last author	
	Gender	Total	Median (IQR)	Total	Median (IQR)
Clinical practice	Female	42	0 (0, 2)	21	1.00 (0, 3)
	Male	43	0 (0, 4)	64	0 [(0, 3)
Editorials	Female	47	0 (0, 1)	26	0 (0, 1)
	Male	105	0 (0, 2)	41	1 (0, 2)
Original	Female	571	2 (1, 5)	353	2 (1, 5)
	Male	706	2 (1, 5)	876	2 (1, 5)
Reviews	Female	17	2 (1, 5)	11	2 (1, 5)
	Male	16	2 (0.75, 3.25)	20	2 (0, 4.250
Overall	Female	677	2 (0, 4)	411	2 (0, 5)
	Male	870	2 (0, 5)	1001	2 (0, 4)

Supplement D

**Table 5:** Proportion of first and last author pairings

First and last author	Number (n = 1411) (%)
Female and female	266 (18.8)
Female and male	360 (25.51)
Male and female	144 (10.20)
Male and male	641 (45.42)

Supplement E

**Table 6:** Proportion of first and last author pairings over time

	Female		Male		Overall	
Publication year	Female (N = 266) (%)	Male (N = 360) (%)	Female (N = 144) (%)	Male (N = 641) (%)	Female (N = 411) (%)	Male (N = 1001) (%)
2007	3 (1.13)	3 (0.833)	2 (1.39)	9 (1.40)	5 (1.22)	12 (1.20)
2008	19 (7.14)	25 (6.94)	7 (4.86)	43 (6.71)	26 (6.33)	68 (6.79)
2009	17 (6.39)	29 (8.06)	10 (6.94)	68 (10.6)	27 (6.57)	97 (9.69)
2010	14 (5.26)	23 (6.39)	10 (6.94)	73 (11.4)	24 (5.84)	96 (9.59)
2011	9 (3.38)	21 (5.83)	6 (4.17)	49 (7.64)	16 (3.89)	70 (6.99)
2012	12 (4.51)	11 (3.06)	12 (8.33)	56 (8.74)	24 (5.84)	67 (6.69)
2013	22 (8.27)	22 (6.11)	10 (6.94)	40 (6.24)	32 (7.79)	62 (6.19)
2014	21 (7.89)	32 (8.89)	16 (11.1)	63 (9.83)	37 (9.00)	95 (9.49)
2015	25 (9.40)	32 (8.89)	13 (9.03)	46 (7.18)	38 (9.25)	78 (7.79)
2016	25 (9.40)	39 (10.8)	12 (8.33)	44 (6.86)	37 (9.00)	83 (8.29)
2017	25 (9.40)	22 (6.11)	10 (6.94)	29 (4.52)	35 (8.52)	51 (5.09)
2018	26 (9.77)	36 (10.0)	9 (6.25)	47 (7.33)	35 (8.52)	83 (8.29)
2019	13 (4.89)	35 (9.72)	12 (8.33)	26 (4.06)	25 (6.08)	61 (6.09)
2020	20 (7.52)	19 (5.28)	8 (5.56)	28 (4.37)	28 (6.81)	47 (4.70)
2021	15 (5.64)	11 (3.06)	7 (4.86)	20 (3.12)	22 (5.35)	31 (3.10)

Supplement F

**Table 7:** Proportion of doctors and dentists registered with the Pakistan Medical Council (PMC) till 2021

Total registered	Male (%)	Female (%)	Overall (%)
Doctors	144,092 (53.1)	127,468 (46.9)	271,560 (100)
Dentists	10,686 (33.5)	21,181 (66.5)	31,867 (100)

## References

[1] Rochon PA, Davidoff F, Levinson W (2016). Women in academic medicine leadership: has anything changed in 25 years?. Acad Med.

[2] Mottiar M (2018). Because it's 2018: women in Canadian anesthesiology. Can J Anaesth.

[3] Silver JK, Slocum CS, Bank AM (2017). Where are the women? The underrepresentation of women physicians among recognition award recipients from medical specialty societies. PM R.

[4] Reed V, Buddeberg-Fischer B (2001). Career obstacles for women in medicine: an overview. Med Educ.

[5] Files JA, Mayer AP, Ko MG (2017). Speaker introductions at internal medicine grand rounds: forms of address reveal gender bias. J Womens Health (Larchmt).

[6] Flexman AM, Parmar A, Lorello GR (2019). Representation of female authors in the Canadian Journal of Anesthesia: a retrospective analysis of articles between 1954 and 2017. Can J Anaesth.

[7] Jagsi R, Guancial EA, Worobey CC (2006). The "gender gap" in authorship of academic medical literature — a 35-year perspective. N Engl J Med.

[8] Miller J, Chuba E, Deiner S, DeMaria S Jr, Katz D (2019). Trends in authorship in anesthesiology journals. Anesth Analg.

[9] Mueller CM, Gaudilliere DK, Kin C, Menorca R, Girod S (2016). Gender disparities in scholarly productivity of US academic surgeons. J Surg Res.

[10] Filardo G, da Graca B, Sass DM, Pollock BD, Smith EB, Martinez MA (2016). Trends and comparison of female first authorship in high impact medical journals: observational study (1994-2014). BMJ.

[11] Silver JK, Poorman JA, Reilly JM, Spector ND, Goldstein R, Zafonte RD (2018). Assessment of women physicians among authors of perspective-type articles published in high-impact pediatric journals. JAMA Netw Open.

[12] Harris PA, Taylor R, Thielke R, Payne J, Gonzalez N, Conde JG (2009). Research electronic data capture (REDCap)—a metadata-driven methodology and workflow process for providing translational research informatics support. J Biomed Inform.

[13] Harris PA, Taylor R, Minor BL (2019). The REDCap consortium: building an international community of software platform partners. J Biomed Inform.

[14] Butler J (1999). Gender Trouble. https://www.taylorfrancis.com/books/mono/10.4324/9780203902752/gender-trouble-judith-butler.

[15] De Cassai A, Correale C (2019). Gender inequality in anesthesiology research: an overview of 2018. Anesth Analg.

[16] Long MT, Leszczynski A, Thompson KD, Wasan SK, Calderwood AH (2015). Female authorship in major academic gastroenterology journals: a look over 20 years. Gastrointest Endosc.

[17] Burton KR, Wong IK (2004). A force to contend with: the gender gap closes in Canadian medical schools. CMAJ.

[18] Barzansky B, Etzel SI (2007). Medical schools in the United States, 2006-2007. JAMA.

[19] Levinson W, Lurie N (2004). When most doctors are women: what lies ahead?. Ann Intern Med.

[20] Phillips SP (2013). The growing number of female physicians: meanings, values, and outcomes. Isr J Health Policy Res.

[21] Ramakrishnan A, Sambuco D, Jagsi R (2014). Women's participation in the medical profession: insights from experiences in Japan, Scandinavia, Russia, and Eastern Europe. J Womens Health (Larchmt).

[22] Wininger AE, Fischer JP, Likine EF (2017). Bibliometric analysis of female authorship trends and collaboration dynamics over JBMR's 30-year history. J Bone Miner Res.

[23] Handelsman J, Cantor N, Carnes M (2005). Careers in science. More women in science. Science.

[24] (2022). ADVANCE: increasing the participation and advancement of women in academic science and engineering careers (ADVANCE). https://www.nsf.gov/pubs/2016/nsf16594/nsf16594.htm.

[25] Zbar A, Frank E (2011). Significance of authorship position: an open-ended international assessment. Am J Med Sci.

[26] Sambunjak D, Straus SE, Marusić A (2006). Mentoring in academic medicine: a systematic review. JAMA.

[27] Masood A (2019). Doing gender, modestly: conceptualizing workplace experiences of Pakistani women doctors. Gend Work Organ.

[28] Ali F, Syed J (2017). From rhetoric to reality: a multilevel analysis of gender equality in Pakistani organizations. Gend Work Organ.

[29] Mohsin M, Syed J (2020). The missing doctors — an analysis of educated women and female domesticity in Pakistan. Gend Work Organ.

[30] Khan N, McGarry K, Naqvi AA, Holden K (2022). The doctor brides. Int J Clin Pharm.

